# Compliance with Clinical Guidelines and AI-Based Clinical Decision Support Systems: Implications for Ethics and Trust

**DOI:** 10.1007/s11948-025-00562-z

**Published:** 2025-11-13

**Authors:** Éric Pardoux, Angeliki Kerasidou

**Affiliations:** 1 Aix Marseille Université, CNRS, EFS, ADES, Marseille, France; 2 IHRIM, CNRS & ENS Lyon, Lyon, France; 3 CNRS, Maison Française d’Oxford, Oxford, UK; 4 The Ethox Centre, Nuffield Department of Population Health,, University of Oxford, Oxford, UK

**Keywords:** Compliance, Trust, Ethics, Clinical decision support systems, Artificial intelligence, Design, Clinical guidelines

## Abstract

Artificial intelligence (AI) is gradually transforming healthcare. However, despite its promised benefits, AI in healthcare also raises a number of ethical, legal and social concerns. Compliance by design (CbD) has been proposed as one way of addressing some of these concerns. In the context of healthcare, CbD efforts could focus on building compliance with existing clinical guidelines (CGs), given that they provide the best practices identified according to evidence-based medicine. In this paper we use the example of AI-based clinical decision support systems (CDSS) to theoretically examine whether medical AI tools could be designed to be inherently compliant with CGs, and implication for ethics and trust. We argue that AI-based CDSS systematically complying with CGs when applied to specific patient cases are not desirable, as CGs, despite their usefulness in guiding medical decision-making, are only recommendations on how to diagnose and treat medical conditions. We thus propose a new understanding of CbD for CGs as a sociotechnical program supported by AI that applies to the whole clinical decision-making process rather than just understanding CbD for CGs as a process located only within the AI tool. This implies taking into account emerging knowledge from actual clinical practices to put CGs in perspective, reflexivity from users regarding the information needed for decision-making, as well as a shift in the design culture, from AI as a stand-alone tool to AI as an in-situ service located within particular healthcare settings.

## Introduction

Digitalization is gaining all spheres of healthcare. Amongst all the new emerging technologies driving what may be a structural shift in healthcare, artificial intelligence (AI) appears to be a cornerstone. AI is a general term which reflects many different technological realities, with machine learning systems and their derivatives being the most promising at the moment (Nittas et al., 2023). AI is becoming prevalent in healthcare applications, and it is poised to play an ever increasing role in the establishment of diagnoses and care plans (Lenharo, 2023). In the case of clinical decision support systems (CDSS), AI systems have (in)direct interactions both with the patients and the clinicians. These interactions in the decision-making processes may happen either in the identification of the pathology, the design of the treatment plans, as well as during the treatment process itself. The implementation of AI is supposed to increase the performances of each of these steps, be it in terms of outcome, speed, cost and so on (Arena, Gaglio, and Vayre 2025).

Notwithstanding the promising abilities of AI to improve healthcare delivery, there is a need to regulate their development, deployment, and integration (Elish, 2018; Sendak et al., 2023). Over the last few years, many guidelines have been issued (Corrêa et al., 2023), while national and supranational bodies around the globe have been developing regulations, such as the European Union’s AI Act. Theoretical discussions producing normative suggestions about how to develop and govern AI-based systems are mainly held within the newly-established AI ethics field. The field of AI ethics encompasses a wide range of disciplines, including philosophy, political science, law, as well as related areas such as machine ethics, computer ethics, and digital ethics (Sætra and Danaher 2022). Existing ethics frameworks produced in the AI ethics field have been criticised for their over-reliance on principles and lack of operability in the real world. High order ethical principles are thus considered hard – if not impossible – to translate into actual rules, policies, and practices. As Mittelstadt (2019, p. 501) summarises: ‘AI ethics initiatives have thus far largely produced vague, high-level principles and value statements that promise to be action-guiding, but in practice provide few specific recommendations and fail to address fundamental normative and political tensions embedded in key concepts’. While there may have been advances in AI ethics since 2019, the crux of the problem persists: there has been no consensus yet on how to best develop “ethical” AI systems. An alternative framing of the problem would be to first ensure the possibility of legal compliance of AI systems before any attempt to render them ethical. Etzioni (2018), for instance, suggests that an AI system should be ‘subject to the full gamut of laws that apply to its human operator’ (31), and Mokhtarian (2018) argues that ‘we likely need to imbue [AI systems] with the capability to comply with the legal systems in which they operate’ (206). ‘Compliance by design’ (CbD) (Sackmann et al., 2008) can be seen as one way of ‘imbuing’ AI systems with the capability to comply with certain explicit rules. Sackmann defines CbD as ‘a preventative approach focusing on the enforcement of desired behavior and the prevention of damaging events’ (ibid., 80). In its legal dimension, the ‘desired behavior’ may be defined according to different legal reference frameworks such as laws, or regulations.

However, others argue that compliance may have other dimensions, beyond the legal one, such as technical or even ethical ones (Pistilli et al., 2023). Reference frameworks are not only laws and policies but can be issued for instance as technical norms (ISO) for technical compliance, and as professional codes or even as self-regulations such as ethical charters in the case of ethical compliance. A process can be labelled as CbD with respect to a specific framework if it is impossible for the complying system to deviate from what is considered permissible by the framework (Sackmann et al., 2008, p. 80).

In this paper, we use AI-based clinical decision support systems as a case study to examine whether CbD with clinical guidelines (CGs) could provide a pathway to building ethical AI systems. We start by describing AI-based CDSS. We then analyse what compliance and CbD might mean in the healthcare context, regarding CGs. Once this background is set, we introduce some of the challenges identified in achieving CbD in AI-based CDSS. We propose an understanding of compliance at the level of the whole clinical decision-making process as a way of addressing some of the challenges posed by CbD with CGs within CDSS. We then propose how changes in the relationship we maintain with technology may help to implement this understanding of compliance in the development of emerging technologies in healthcare and beyond. We conclude by discussing the implications of compliance for ethics and trust regarding emerging technologies.

## AI and Clinical Decision-Making

The introduction of AI technologies in healthcare comes with uncertainty in terms of medical, social and ethical outcomes. Regarding medical impact, calls for the introduction of AI is often accompanied with promises of a paradigm shift in health and care practice, using AI ‘to create learning systems that can readily convert data into actionable possibilities’ (Hood et al., 2022, p. 40). Proponents of AI and big data such as Leroy Hood and his colleagues suggest they will redefine our understandings of diseases and pathologies, driving care towards a more predictive endeavour, enabling ‘early-stage reversal of disease processes’ (ibid., p.38). AI-based healthcare is thus inheriting and renewing a long history of promises linked to predictive medicine (Olivier, 2021). However, some have argued that such promises may be overhyped or even based on false assumptions^1^, reflecting more economics of promises rather than pragmatic predictions (Arena, Gaglio, and Vayre 2025). Regarding societal impact, AI could lead to the re-distribution of tasks and roles between patients and physicians calling for a reappraisal of established practices and responsibilities. Ethically, such systems may perpetuate or even amplify pre-existing problems in healthcare. For example, in the allocation of care resources (Obermeyer et al., 2019), or with the exercise of patient-centred empathetic care (Kerasidou, 2020). Understanding how to harness the benefits of AI while limiting possible harms is not yet clear. In this context of uncertainty, one way to safely deploy emerging AI-based systems would be to ensure that they are developed according to norms and values relevant and applicable to the sociotechnical contexts in which they will be integrated, before control through regulation becomes too difficult to implement (Collingridge, 1980). Meeting this challenge requires an understanding of how CDSS are developed. This would facilitate the learning process of how to better evaluate the promises, and mitigate or avoid potential problems during the integration of new AI systems and models within them – especially those based on machine and deep learning.

Attempts to integrate AI in CDSS is not a new phenomenon. To a large extent, the development of CDSS goes hand in hand with advances in AI. Although there is a large variety of AI methods and strategies, there are two main approaches at building AI models: the so-called symbolic and connectionist approaches. While the symbolic approach relies mostly on rules, the connectionist approach builds models from data providing solely inputs and outputs—except in the case of unsupervised learning where only inputs are provided—the learning process then yields a program, modelling relations between inputs and outputs. Before the boom of machine learning in the 2010s, CDSS relied mostly on the symbolic approach, epitomised by expert systems embedding rules and explicit medical knowledge (Musen et al., 2014)^2^. Such systems poorly succeeded in integrating routine medical practices due to their lack of consideration of users’ needs and expectation (Heathfield, 1999). These difficulties also illustrate the overall complexity, if not the impossibility, of eliciting and formalising expert knowledge (Collins, 1990). Machine learning and mining techniques, both leveraging big data, have fundamentally changed the field of CDSS and will probably continue to do so (Sittig et al., 2016). CDSS can now be trained on large amounts of actual clinical data, backing or substituting previous knowledge-based systems or expert systems based on limited data. Their functionalities depend on the extraction of patterns or implicit structures from datasets. Such an approach was firstly used in highly specialised diagnostic tasks, such as medical imaging, radiology paving the way (Pesapane et al., 2018). However, CDSS are now evolving into more multimodal systems, integrating health data from various sources (in a multi-omic fashion), in order to contribute to diagnoses and treatment predictions (Nittas et al., 2023). Recently, some experts in the field have been calling for developing highly flexible foundation models for medical AI, enabling General Medical AI (Moor et al., 2023). Medical foundation models remain a goal that is not attainable in the short term. However this provides a future horizon for CDSS. In this emerging trend, there is an increasing demand to train AI models on a broad spectrum of sources. This diversification of data sources goes beyond conventional clinical datasets centred on narrow families of pathological conditions. Electronic health records, including medico-administrative information, become a source of pattern identification in clinical pathways. Systems trained on such data may be used for instance to help design treatments, identify pathologies, or even repurpose drugs. In the context of this paper, we only consider CDSS based on offline AI models, providing assistance in clinical decision-making. Such models already offer significant challenges. They are susceptible to produce faulty results, for instance, when applied on cases from sub-populations not sufficiently represented in their training dataset (Koçak et al., 2024). Furthermore, to the extent of our knowledge, AI-based CDSS cannot recognize the limits of their expertise nor do they incorporate implicit knowledge (Gillies & Smith, 2022). This implies AI systems users should be aware of ‘the extent to which they may legitimately rely on the outputs from machine learning programmes’ (ibid., p.46). These concerns notwithstanding, AI-based CDSS are presented as a promising addition to the toolbox of medical decision-making, to ‘augment the ability of health-care providers to improve patient care’^3^. In this paper we do not wish to challenge the soundness of the promises of AI-based CDSS. Instead, we take the potential of such CDSS to improve clinical practice and patient care as a working hypothesis. Our aim is to examine whether CbD with CGs could mitigate some of the risks identified in these systems, thereby establishing if it could become a requirement for AI-based CDSS. This calls for a thicker definition of compliance and for the identification of how CbD with CGs could or even should be implemented in AI-based CDSS.

## Compliance, Healthcare, and Clinical Decision-Making

Before going into the challenges associated with making AI-based CDSS compliant by design, we propose a brief analysis of the concept of compliance. Compliance is a multi-faceted term, even in domain-specific uses (Presti, 2021). Historically, it was first used to refer to the compliance of products and services with applicable laws, rules and governing norms (Miller, 2014). Nowadays, compliance is a term often used in organisations, particularly in finance, to mean ‘the set of rules, procedures, bodies, and offices in charge of managing a particular operational risk: the risk of *incurring judicial or administrative sanctions*,* significant financial losses*,* or reputational damage as a consequence of violations of mandatory rules (laws or regulations) or self-regulatory rules (e.g.*,* statutes*,* codes of conduct*,* self-regulatory codes)*’ (Presti, 2021, p. 25, emphasis by the author). When it comes to healthcare, compliance has a multiplicity of meanings and does not only refer to the set of laws and regulations medical professionals and institutions need to follow. For instance, the term compliance has been used to describe the adherence of patients to treatment plans (Evangelista, 1999)—although the use of the term in this context is controversial and now widely disused (Tilson, 2004). It can also point to corporate compliance within healthcare institutions to avoid fraud and abuse (Guinn, 2000). Lastly, compliance is also used regarding CGs and protocols for diagnosis and treatment (Oliart et al. 2022). It is this meaning we will discuss mainly in the remainder of the article as it is the most relevant to CDSS (see section “Challenges to CbD in developing CDSS”).

Compliance, be it in finance or in healthcare, is in every case a relational notion. The complier is subject to normative expectations external to their autonomy, while being able to enter into a dialogue about how to change such expectations (Hess, 1996; Chandler, 2014). Compliance can either be static or dynamic. Static compliance is the process of checking if each action aligns with normative expectations – this is also known as ‘conformance-checking’ or ‘compliance by detection’ (see below). Dynamic compliance signals that the complier is in a situation where, in addition to static compliance, improvements can or must be made thanks to what is learned from the environment. Such changes can come from the complier or from an evolution of the normative expectations. Compliance programs in corporations can thus act in two ways: detecting compliance defects, and improving the inner workings of the organisation (Sackmann 2008). There are two main strategies to achieve compliance: ‘compliance by design’ and ‘compliance by detection’ (Sackmann 2008). The former reflects the intention to prevent any risk from occurring, the latter is retrospective, enabling possible *ex post* mitigation or subsequent prevention. None of these approaches can operate alone, as the ‘by design’ approach requires elements of detection, whilst capabilities of detection need to be implemented through the design of the system. Nonetheless, these strategies represent the two main ways of dealing with risk.

CbD as a strategy can be useful in contexts exhibiting high-risk and low tolerance to non-compliance. Healthcare is a high-risk area: even when cases are not concerning life-or-death situations, medical decisions always often pertain to the quality of life of the patient. Non-compliance can also be problematic in healthcare. For instance, prescribing penicillin to an individual who is allergic to penicillin is a direct source of harm due to non-compliance with best practices – and with common sense. Beyond this example, one may question whether strict CbD with best clinical practices is desirable for the outputs of AI-based CDSS. Low tolerance in the clinic to AI-based recommendations of suboptimal practices would motivate developing these CDSS as compliant by design with best practices. However, with what should they comply exactly? How could compliance be thought about and possibly integrated by design in the case of a CDSS trained on electronic health records and past medical data? How would best practices be defined in such cases?

## Challenges to CbD in Developing CDSS

Evidence-based medicine is the current dominant paradigm within allopathic medicine. It asserts that best practices in clinical care should be based on the best possible evidence (Sackett, 1997). This can be disseminated in the form of CGs that frame what is appropriate when it comes to medical practices (Gatta et al., 2019). Legally, it has even been argued that CGs could be used to establish the standard of care in medical litigation (Samanta et al., 2006). CbD in the case of clinical decision-making could therefore be conceived with respect to CGs based on high quality scientific evidence, including systematic reviews, meta-analyses and randomised controlled trials. Compliance of clinicians with guidelines has already been shown to improve as a result of the use of CDSS and computerised guidelines, as long as the digitalisation is supported through active promotion and consensus (Williams et al., 2004; Morgan, Goodson, and Barnett 1998). Patients can also play an active role in improving overall compliance with CGs. For example, granting patients access to their health records in order to keep it up-to-date can positively influence compliance with CGs in the planning of care by clinicians (Staroselsky et al., 2006). For AI in healthcare, the challenge is to examine whether CbD with CGs should be a requirement for emerging AI-based CDSS.

In the current state of the art of CDSS, verifying compliance of clinical practices with guidelines relies mostly on conformance checking, which is part of the ‘process mining’ field (Oliart, Rojas, and Capurro 2022; Grüger et al., 2022). In order to perform conformance checking, it is first necessary to define reference process models based on guidelines. Subsequently, actual clinical decisions would be modelled in a similar manner and then compared to the reference. Conformance checking is thus dependent on static references, which need to be updated as often as guidelines and medical consensus change (Oliart, Rojas, and Capurro 2022). Such an update system linking new CGs with already implemented CDSS is technically conceivable, but not yet developed. In addition to the necessary accessibility, machine-readability and updatability of the conformance-checking system of the CDSS outputs, another issue rises, which is not technical but medical: are CGs sufficient for orientating practices in healthcare?

Guidelines are not a panacea and there are many reports of clinical practices deviating from them (Barth et al., 2015). Basic medical knowledge may contradict guidance provided by CGs (Spiotta et al., 2014). Some situations are also not covered by CGs. An example of this is patients with multiple morbidities, where the treatment of one condition may result in adverse effects due to comorbidities (Hughes et al., 2012). One should thus not equate CGs with universal guidance about how healthcare should be practised (Djulbegovic & Guyatt, 2014). However, CGs remain a compass to improve care, especially in the digital age. As Mehl et al. (2021, p. e215) argue, there is a need for ‘a systematic, transparent, and testable pathway from narrative global guidelines to localised digital systems’, reflected by the World Health Organisation SMART guidelines. In this context, global CGs are considered both useful for overall guidance and insufficient for a complete determination of practice. A margin of indeterminacy remains between the global CGs and their contextual integration.

Another challenge arises when considering how to develop CDSS compliant by design with CGs. This is illustrated by the transition from specialised CDSS (designed to perform well on a narrow set of applications) to general all-purpose CDSS potentially based on AI. The multiplication of clinical applications a single CDSS can be used for directly implies the multiplication of CGs that need to be taken into account. This is a significant technical problem. With current best techniques, when compliances of clinical processes with different CGs are measured, only a few sections of individual guidelines are considered (Oliart, Rojas, and Capurro 2022). In this context, implementing large-scale compliance is a challenge.

One way to address the challenge presented by trying to implement large-scale compliance is to widen our understanding of CbD through the distinction of *ex ante* and *ex post* compliance strategies. So far, we have only considered *ex post* conformance checking, after the result of the CDSS was obtained. Such conformance checking by computers enables compliance in the form of compliance by detection (Sackmann et al., 2008): by verifying and then pointing out at irregular cases *ex post*, actions can be subsequently undertaken to mitigate such practices. In the case of CDSS, such compliance by checking can become CbD, if this verification step is included directly within the system, before displaying results to the clinicians. Compliance as a conformance checking mechanism could be an instantiation of ‘medical verifiers’ of AI decisions—in a similar fashion to the Etzionis’ idea of AI guardians (A. Etzioni and Etzioni, 2016).

An alternative to internal *ex post* compliance checking would be to hypothesise that training *ex ante* AI-based CDSS only on compliant data would result in mainly—if not exclusively—compliant outcomes in the predictions of the algorithm. CbD would thus be reached as only compliant cases were used. There are at least two bottlenecks to this strategy. Firstly, databases of compliant-only medical data do not exist at the moment to the best of our knowledge. Secondly, depending on their design, there is no guarantee that such models would not make things up, as large language models do (Zhou et al. 2024)^4^. Therefore, relying on the *ex ante* approach only, would not be appropriate. *Ex ante* training and *ex post* verification approaches would of course be possible to hybridise. CbD in AI-based CDSS would probably require a knowledge-based approach, leveraging CGs and medical knowledge^5^ to check *ex post* the outcomes of the system. However, CGs and medical knowledge may be contradictory and do not cover all possible cases (Spiotta et al., 2014; Hughes et al., 2012; Djulbegovic & Guyatt, 2014). Building AI-based CDSS following such a knowledge-based approach may inherit the non-coherent and non-exhaustive characters of CGs and medical knowledge. Compliance with both CGs and medical knowledge would exclude outcomes of the CDSS that are compliant only with CGs and not with medical knowledge or the other way around. Such a view relies mostly on a static understanding of compliance, inherited from process mining and aiming at checking all the boxes. Alternatively, CDSS could offer the possibility to reflect the diversity and sometimes ambiguity of medical knowledge and CGs. For instance, non-compliant but optimal CDSS outputs could be provided along with the discrepancies identified regarding normative frameworks – be it CGs or medical knowledge. This may be exemplified by a machine learning based CDSS that is trained on data from clinics where a novel treatment recommended by guidelines is not yet available^6^. As long as this novel treatment is not available, full compliance with guidelines is not reachable both for clinicians and for the outputs of the AI-based CDSS, since its training data from the local clinic does not include cases using this treatment. Nonetheless, the CDSS may still give useful advice for locally best suited options. Displaying non-compliant outputs of the CDSS while highlighting the reasons of this non-compliance could prompt users to investigate the soundness of these outputs. Reflexivity and potential changes in the clinical decision process could hence be fostered – including infrastructural changes, such as gaining access to the new treatment recommended by the CGs. This would require compliance to be considered more widely than in internal *ex ante* and *ex post* compliance strategies to produce only compliant outputs with the CDSS. In the next section we reconsider the question of the CbD of CDSS with CGs by situating compliance as a broader program concerned with the entire clinical decision-making process.

## CbD as a Situated Process in Healthcare

In his introduction to corporate compliance, Miller (2021) introduces the idea that there have been two historical phases to compliance. ‘Compliance 1.0’,according to Miller, is characterised by ‘the mindset of a bookkeeper or auditor who defines their job as checking off items on a list’ (ibid. 3), thus very close to the depiction of compliance as conformance checking discussed above. ‘Compliance 2.0’ is the modern paradigm of a deep integration of compliance in the agile functioning of contemporary corporations. It accomplishes ‘a melding of top-down strategies that draw on generalizations and abstract principles and bottom-up strategies that implement lessons learned from past successes and failures’ (Miller, 2021, p. 4). Conformance checking in this context is just a static feature of a larger and dynamic compliance process, aiming at improving the system in which it is performed. Compliance 2.0 is about dealing with the multiple different norms (legal, medical, cultural…) weighing upon decision-makers. Compliance should therefore be assessed as a ‘string of reiterative processes that occur in their situational context’ (Wu & Rooij 2019, 581). Thus, compliance can be thought as dynamic and situational, instrumental to improvements in the system process. As compliance is relational, it cannot be embedded in a stand-alone fashion within a non-contextual ‘one-size-fits-all’ tool.

Translated into the case of AI-based CDSS, what would an understanding of compliance as a dynamic and situational process imply? Healthcare is not a purely technical domain. Compliance of CDSS outputs with CGs—even free from the above-mentioned defects—would not be sufficient to fully determine best suited clinical decisions. Other elements, such as the carer’s experience, the patients’ preferences, the hospital facilities or the institutional and bureaucratic contexts have to be taken into account. Also, the local clinical context is important, as regional or national healthcare systems can lead to varying practices, habits and administrative routines in the clinic (Cicourel, 1990). Patients, clinicians, healthcare institutions: each of these entities may have their own social, ethical, political, technical and scientific backgrounds. All these dimensions impact on the way these stakeholders envision AI systems and engage – or not – with them. Furthermore, their design and functionalities reflect the intentions, aims and goals of the technology designers and producers. Surveying the introduction of AI within medical decision-making procedures invites us to consider the designers and producers of AI systems as additional actors and stakeholders. Compliance with CGs is thus one dimension amongst many that need to be taken into account when developing a CDSS. The outcomes obtained from the integration of CDSS should not be judged in the abstract conditions of research and development laboratories but rather in the sociotechnically rich contexts of the clinic with its various stakeholders.

Some of the enablers of a sociotechnically-minded integration of CDSS can be implemented at the technical levels within the CDSS in order to help reaching CbD with CGs at the sociotechnical level – and not only at the level of the AI-based CDSS outputs. For instance, designing the interface of the CDSS in order to give outputs only if there has been a first clinical input from the clinician could avoid partly automation biases (Goddard et al., 2012), as well as being in line with the way expert critiquing systems are conceptualised (Groot et al., 2009). Alerting users about non-compliant outputs of CDSS, instead of censoring them by design could also be a way to inform clinical decisions whilst avoiding potential clinical conservatism. AI-based CDSS can thus still be used to support shared decision-making in the clinic. In this context, checking and displaying compliances with CGs could help improve the shared clinical decision-making process. It is not the CDSS in itself that has to be fully compliant. Rather, it is the overall decision-making processes that should be compliant, with AI as one type of support tool amongst others. Here, compliance does not mean conforming with standardised practices. Instead, it means that the output should fit into the overall improvement process of clinical care. Patients and clinicians would still be able to decide for non compliant care procedures, as it is already the case. However, the CDSS could provide support as to why such procedure could be well-suited, even if not compliant. For instance, if the CDSS is trained on past medical data, explainable outputs could point to previous similar cases, which would enable the discussion of neighbouring cases amongst experts. It could also indicate published studies on the matter (Lebedev et al., 2020) Research on CDSS combining these different kinds of reasoning—knowledge-based from guidelines and medical literature, data-driven, and explainable methods—is already ongoing (see for instance Kovalchuk et al., 2022).

Our main point lies in the call for a systemic view of compliance, taking the whole sociotechnical process of clinical decision-making into account. Thinking of AI-based CDSS as vectors for CbD in healthcare would thus be a support for continuous learning through group reflection (Bucalon et al., 2023). Such systems could also contribute to healthcare improvement if they go beyond the sole focus of their individual effectiveness (Dixon-Woods, 2019) Dixon-Woods indeed argues that there is ‘a need to establish improvement [in healthcare] as a collective endeavour’ (ibid. p. 3) by shifting from a “product dominant logic” to a “service dominant” one (ibid. p. 2). AI-based CDSS would thus provide a basis for evidence-based research of future choices made in the clinic, at the condition they can implement some needed features.

## Sociotechnical Conditions to Implement CbD in Healthcare

Thinking of compliance as situational and dynamic requires us to take a different attitude towards the integration of emerging technologies in healthcare, such as AI. Clinical decision-making is not a fixed-case scenario with a one-size-fits-all answer. In design, as in healthcare, problems and solutions co-evolve. Improvements of clinical decision-making processes should be an exploration of the problem and solution spaces conjointly, not solely of the solution-space nor of the problem-space (Smith et al., 2023). Locating compliance in the design of the CDSS amounts to focusing solely on the technological solution-space, ignoring its social integration, as well as the sociotechnical context where the problem emerged. What might be thought of as a solution in the initial idea of a project might be seriously complicated by practice. CDSS may not be user-friendly, they could slow down healthcare practitioners, disrupt clinical workflows, display useless recommendations or provoke alert-fatigue (for a review of obstacles to CDSS integration see Meunier et al., 2023). Hence, there is a need to think dynamically about the relations between the CDSS, its social context which they emerge from, and the goals its implementation is supposed to achieve. The main question leading the experimentation with and integration of an AI-based CDSS in the clinic should be ‘Is this AI-based CDSS actually solving the problem it is set out to solve?’. What makes CDSS useful in practice therefore exceeds their mere compliance with CGs and other reference frameworks. Implementing compliance-readiness might only be a first step towards improving clinical practices. It calls for reflexivity and participation of all stakeholders in the design of clinical practices that AI-based CDSS may be a part of. Accepting the dynamism and situatedness of clinical decision-making implies acknowledging the intrinsic uncertainty built into the various elements participating to the process (Hofmann 2022). CDSS should foster the ability to constantly update and challenge established medical knowledge and practices. As for CGs, AI-based CDSS need to enter the ‘endless task’ of constant updating (Baron et al., 2017). This calls for AI-based CDSS to be easily updatable (Peleg, 2013; Martin et al., 2020). For the CDSS system to actually improve clinical practices through the compliance process, it needs to be flexible enough to be redesigned by users according to their needs—this requires users to have a sufficient understanding of how the system functions. Implementing compliance programs should not be done in order to blindly execute predetermined sequences of action, but rather as a way to define a ‘comfort zone’ (Pérezts & Picard 2014). Although this concept was not developed in a clinical context, its import in the articulation between compliance with CGs and biomedical ethics could be fruitful. Pérezts and Picard describe comfort zones as situations that ‘allow the creation of a cognitive sphere where several and conflicting injunctions can be confronted to ultimately allow the maintenance and the evolution of certain logics’ (ibid., p.849). Translated into clinical decision-making aided by AI systems we posit that displaying the compliance of CDSS outputs with normative frameworks such as CGs, medical knowledge, or even legal norms, could help create these comfort zones. This anticipates the pro-ethical approach we present in the following section. Performing compliance should thus be seen not as a good in itself but rather as a means to fostering other ethical and professional values, such as trust and patient-centeredness, while increasing the overall reliability of the process.

The sociotechnical conditions of implementation of CbD programs imply a shift in the way emerging technologies are framed in society: instead of omnipotent solutions, they should rather be considered as experiments posing new problems. As Ibo van de Poel suggests, the introduction of a new technology in society can be thought of as a moral experiment (van de Poel, 2017). Explicitly calling emerging technologies experiments—whatever their scale—can pose problems in terms of acceptance. However, in the long run, a cultural shift towards an acknowledgement of the experimental and uncertain nature of emerging technologies could enable a better understanding of what is at stake in sociotechnical changes provoked by AI (Henriksen & Olesen, 2023). Building CbD programs for AI CDSS should thus be done while recognising that such systems are experimental both regarding their technical and moral characteristics (van de Poel, 2017). These characteristics are bound to evolve through the effect of the compliance program. Problems and solutions experimentally co-evolve, the same way that our moral and epistemic understandings of health, illness and disease also do.

## Compliance, Trust, and Ethics

In the introduction, we started from the suggestion that CbD could be a possible alternative to the issue of encoding ethics within AI systems. How does this stand after our inquiry? As it has already been shown elsewhere (Michaelson, 2006), ethics does not equate with compliance with external codes, but it is also about balancing conflicting principles and values. CGs only represent one set of values amongst others. Complying with CGs cannot be assimilated to the ethical provision of care as it can be at odds with patients’ values for instance. However, building a compliance infrastructure that enables quality improvement and critical reflection on care practices can be seen as a way to foster reliance on AI-based CDSS, and even trust in healthcare professionals and systems. Pro-ethical design, as Floridi (2016) puts it, is the shaping of only the information about what the actual options are. Disclosing all the clinical options suggested by the AI-based CDSS, in association with their respective compliances^7^, is in line with pro-ethical design. AI-based CDSS can be used to inform on all the considered options, and not enforce a particular one, nor reject options ab initio because they would not be compliant with current CGs for instance. Empirical studies have also suggested that when it comes to trust in AI tools used in their care, patients are concerned with the extent to which such tools can promote ethical values that matter most to them, and support patient-centred care (Dlugatch et al., 2023). Clinicians seek reliability from AI-CDSS in order to feel confident in incorporating them into their clinical decision-making (Dlugatch et al., 2024). It is important, therefore, to depart from approaches based on the non-tolerant and paternalistic shaping of possible actions such as structural nudging, opt-out choice architectures or CbD performed strictly following CGs and medical knowledge. Displaying non-compliant outcomes of the CDSS (along with the relevant information about why they are not compliant) would grant stakeholders the possibility to critically assess the potential medical interest of non-compliant outcomes, enabling a more value pluralistic and patient-centred pro-ethical design. The clinician would be able to ponder whether being non-compliant with guidelines is appropriate, especially regarding other values at play, including patient values. Conversely, the patient would acknowledge the potential risks and non-compliance of the various options on offer. The care institution would be able to reduce negligence, by being fully informed about the evolving standards of care. Risks of negligence (Schönberger, 2019) could thus be reduced. The CDSS would no longer be only a resource tool for decision-making but it could provide a platform for stakeholders to make clinical decisions. Thanks to interoperability, tracks of decisions could be stored to improve processes within the clinic and between different centres. Including an AI-based CDSS within a new healthcare setting does not only requires paying attention to technical characteristics, such as flexibility or updatability (see section “Challenges to CbD in developing CDSS”). It also requires continuous monitoring in order for new knowledge to be produced, captured and evaluated alongside the integration of the emerging technology within a novel sociotechnical context (Gaglio & Loute 2023; Henriksen & Olesen 2023). All stakeholders should also be involved in the monitoring and evaluation process, that would ascertain the successful (or not) implementation of the new technology. In the case of CDSS, the aim of the implementation could be to improve the overall quality of care. This would require flexibility both from the clinical context of integration and from the CDSS. Designing AI-based CDSS to be CbD-ready equates with rendering them suitable to constant updating.

Our discussion of compliance has focused primarily on compliance with CGs. This could be considered as an epistemological compliance since complying with CGs could equate with the assurance of having a consensual rationale guiding medical decisions. There are other kinds of normative frameworks one can comply with: legal texts, ethical codes or deontological charters (Pistilli et al., 2023), that can also be action guiding. Achieving compliance with CGs should not be considered as value-free or as normatively neutral. As Bart Molewijk and his colleagues underlined, ‘there is no value-neutral (framing of) information, patients will always be manipulated both by the value-laden nature of information itself and by the way doctors (consciously or unconsciously) provide information’ (Molewijk et al., 2003, pp. 83–84). There is no reason to believe the presentation of information by AI-based CDSS would escape this fact. Our claim is that by providing references to CGs or to medical knowledge, such systems can normatively contextualise their outputs. This can be done by being explicit about what CDSS or CGs try to optimise through their recommendations (Molewijk et al., 2003). For instance, is it maximum life expectancy, quality-adjusted life expectancy, or something else? CDSS should by no means be surrogates for critical and ethical thinking by the individuals themselves – be they healthcare practitioners, patients, or hospitals managers. Shared decision making in medicine is the encounter of various systems of values that may clash – even internally. Disclosing the compliance of outputs from AI-based CDSS is only a way of trying to disclose normative assumptions behind aids to decision-makers. As already mentioned, one should not equate evidence-based decision-making and ethical decisions. CGs are only a global compass stakeholders need to localise and contextualise (Mehl et al., 2021). Blindly following the outcomes of a CDSS would not ensure that ethical values, principles or virtues have not been violated. The value of displaying compliance with CGs as we described can thus be considered as prudential – as it could inform decision-makers – but it does not constitute a good in itself.

We proposed that thinking of compliance with CGs as ecosystemic rather than exclusively situated within the AI system, in a CbD fashion, could facilitate ethical reflection in the overall clinical decision-making process. Further considerations on how to implement such a sociotechnical CbD need to be made. For instance, would introducing a strong compliance program only entail a reorganisation of current clinical decision-making processes or would there be a need for a new role, in charge of the overall compliance and ethics assessment, akin to compliance officers in large business corporations? What would be the functions and craftsmanship of such ethics & compliance experts? Conceiving healthcare systems and organisations able to aptly enact ethics and compliance is thus largely a work in the making, as is compliance itself. Full compliance with CGs is thus an ideal: always pursued, never totally secured, and constantly reassessed and re-evaluated.

## Conclusion

The ethics of emerging technologies such as artificial intelligence is a conundrum for regulators. Some authors, including Etzioni and Mokhtarian advocated for building compliant by design AI systems with laws and regulations. In the case of healthcare, such an approach would imply for AI-based CDSS to keep up with the constant evolution of clinical guidelines. Compliance understood as the mere conformance checking of AI-based CDSS outputs with CGs would not perform such a task. However, conceiving compliance at a larger scale than within the CDSS only could indeed transform it into a driver for better and more ethical decision-making processes. Enabling compliance programs to improve the quality of care requires sociotechnical conditions that would be beneficial to trustworthiness and ethical reflections in the clinical decision-making process. Compliance with CGs should not be seen as an end in itself, it is rather instrumental to the improvement of decision processes in healthcare, in a sociotechnically enriched setting. Methodologies enabling this implementation have yet to be explored and described. The mere checking and displaying of compliances with CGs would not be sufficient. Indeed, there are potential pitfalls in the design of the human-CDSS interface: cognitive overload and alert fatigue should be avoided, affordance should be high to enable a low entry cost to the system, distribution of responsibilities after implementation should be anticipated, and so on. Social sciences, humanities, and ethics have undoubtedly a role to play in the process of uncovering the best ways to design AI-based CDSS. These systems should be able to check compliances of their outputs with CGs while also being beneficial when integrated in the sociotechnical fabric of medical and clinical contexts. These practical and methodological considerations are beyond the purpose of this article. However, our argument has concrete implications on how AI-based CDSS and their infrastructure should be designed. Firstly, AI-based CDSS should be equipped with ways of checking compliances of their outputs with human-understandable normative frameworks, such as CGs. Secondly, CGs should be findable and readable both for humans and CDSS. Thirdly, the clinical decision-makers should be put in a context where a critical assessment of their options is made possible, especially when these options are advised by an AI-based CDSS which compliance with CGs was not totally complete. Fourthly, integration of AI-based CDSS in the clinic should be made whilst acknowledging its experimental dimensions, such as what it aims to improve and how it should be assessed. These conditions could be seen as a necessary (albeit non sufficient) basis to design AI-based CDSS that are pro-ethical, i.e. that foster a sociotechnical context that allows for an ethical approach to experimenting, learning, and improving medical decisions.

## Data Availability

Not applicable.

## Footnotes

1 On this matter, we thank an anonymous reviewer for drawing our attention to Delphine Olivier’s book. The fourth part especially deals with the limits of current goals of predictive medicine, which cannot yet prove that individual health is fully predictable and calculable.

2 As one anonymous reviewer aptly pointed out, expert systems do not only rely on rules nor on symbolic approaches. Some were developed for instance as probabilistic systems.

3 Here we quote the World Health Organisation report on *Ethics and Governance of Artificial Intelligence for Health: WHO Guidance* 2021 (page *xi*) as a token of what appears to be the supranational consensus surrounding the integration of AI technologies in healthcare.

4 As one anonymous reviewer suggested, it is important to recall that not all AI systems are prone to so-called ‘hallucinations’. However, the development of CDSS is increasingly relying on large language models to interpret electronic health records, CGs, medical knowledge sources, and so on. In compliance strategies it can also be seen in the recent development of Retrieval Augmented Generation, which can produce unsound results (‘hallucinations’) when based on large language models.

5 In this context, medical knowledge can be understood as the information required to interpret and apply the contents of CGs, such as definitions of concepts and causal mechanisms.

6 This is illustrated for example by an article by Lee et al. (2024), in the case of hepatocellular carcinoma (HCC). Machine learning models trained on past data provided useful recommendations for individual cases, even in advanced stages of HCC where treatments recommended by guidelines were never recommended. This is explained by the fact that these recommended options were absent from the training dataset, thus rendering the ML model unable to propose them as treatment alternatives. This absence is due to the clinical context as these treatments were not employed yet in the clinics where the model was trained and used.

7 The use of the plural is here intentional: one could design the system according to various reference frameworks which are not necessarily consistent. Compliance can thus be gauged either globally, or in a pluralistic fashion, against different frameworks—non-consistent CGs, environmental norms, and so on.

## Declarations

### Rights Retention Statement

This research was funded in part by The Wellcome Trust, 203132/Z/16/Z. For the purpose of Open Access, the author has applied a CC BY public copyright licence to any Author Accepted Manuscript (AAM) version arising from this submission.
